# Perceptions and Attitudes of Jordanian Medical Students on Using 3D Interactive Anatomy Dissection in Teaching and Learning Anatomy

**DOI:** 10.2147/AMEP.S419333

**Published:** 2023-08-03

**Authors:** Khaled Funjan, Laith Ashour, Muna Salameh, Ayman Mustafa, Mohammed Seed Ahmed

**Affiliations:** 1 Department of Basic Medical Sciences, Faculty of Medicine, Al-Balqa Applied University, Al-Salt, Jordan; 2 Department of Basic Medical Sciences, College of Medicine, QU Health, Qatar University, Doha, Qatar

**Keywords:** Anatomage table, cadavers, medical education, virtual anatomy dissection

## Abstract

**Background and Aims:**

This study evaluates the use of virtual anatomy dissection (Anatomage Table) in teaching anatomy for Jordanian medical students. The study also highlights any gender differences in students’ perception on this method of teaching anatomy.

**Methods:**

This is a cross-sectional questionnaire-based study that was carried out on medical students enrolled in Al-Balqa Applied University, a Jordanian public university. A group of expert anatomists designed a questionnaire that investigates the students’ perceptions and attitudes toward using virtual anatomy dissection. The questionnaire also investigated student’s opinions and expectations on the impact of using this method on the academic achievement of students.

**Results:**

The findings of the study showed that most students agreed that Anatomage Table helped them better understand (64.3%) and memorize (64%) anatomy lectures. In addition, most students were interested in using this learning method in lab groups (72.3%). However, the didactic approach that combined anatomical models and the Anatomage Table was preferred over the unilateral approach that included only the Anatomage Table (80.5% vs 30.2%, *p*<0.001, *r*=0.9). Of note, there was a statistically significant difference between males and females in their preference for Anatomage Table (*p*<0.001), and in their perceptions on the impact of Anatomage Table on understanding of lectures (*p*<0.001) and memorization of anatomical structures (*p*=0.004).

**Conclusion:**

The Anatomage Table is a powerful teaching and learning method in undergraduate medical education. Its application to Al-Balqa Applied University has proven to be effective so far. It can be used to overcome the problems facing anatomical education in the college of medicine in Al-Balqa Applied University and perhaps other universities in Jordan, but this needs better cooperation between universities and stakeholders to provide adequate funding for this method.

## Introduction

The preclinical knowledge that all medical schools provide to their students is gained through basic medical sciences. Of which, anatomical sciences make a substantial share of medical curricula. This is because medical practitioners must have sound knowledge of the human body anatomy to conduct physical examinations, evaluate radiologic investigations, operate surgeries, and perform other medical interventions.^1–3^ The value of anatomy education to clinical training of medical and paramedical students triggered the vertical integration of anatomical sciences with clinical training.^4^,^5^ Moreover, anatomical sciences provide medical students with a reservoir of medical terminology that applies to all other medical sciences.^6^ Such value of anatomical sciences demands continuous efforts toward adopting innovative teaching methods to make human anatomy a student-friendly subject.^7^

Anatomy teaching has relied on cadaver dissection since its beginning. Until today, many reports still acknowledging the virtues of hands-on dissection and its importance in learning and teaching anatomy.^8^ On the other hand, other reports support the inclusion of new educational technologies to replace cadaveric dissection.^9^ In fact, it is unclear whether to keep utilizing cadaveric dissection as a teaching tool or to substitute it with non-cadaveric methods that make use of the current state-of-the-art technologies.^10^ One can see that both sides of this argument are valid after carefully considering it. Cadavers are great for exhibiting a variety of typical anatomical variations, explaining the anatomy of major organs, and seeing the 3D body structure, thus providing clinically relevant anatomical information.^11–13^ For this reason, lots of researchers and academics consider keeping “hands-on” cadaver dissection essential and crucial for anatomy education.^14^,^15^ However, dissection might have some limitations and shortfalls. For example, learning topics like surface anatomy, the anatomy of small inconspicuous organs, nerves, veins, lymphatics, and so on is ineffective.^11^ In addition, some students experience worries and anxiety because of the dissecting rooms.^16^ Many researchers, instructors, and even students believe that employing dissection alone is insufficient to understand anatomy and that adding other educational techniques is advantageous or even needed.^11^,^17–19^ Many medical schools have suspended dissection, regardless of which side of the debate they believe in. This was done for variety of logistic reasons, including the growing number of medical students, the limited time given to anatomy in integrated medical curricula, and most importantly the shortage in cadavers and qualified staff.^20^ In fact, the availability of other convenient tools, such as plastic and plastinated models, provided appropriate substitutes.^6^

Medical education has advanced dramatically in recent years. The use of computer-based and multimedia-assisted educational aids like movies, animations, 3D models, and virtual microscopes is prominent in the field of anatomy.^21^,^22^ This was supported by positive feedback reported by many authors who investigated the impact of using such tools when teaching anatomy to medical students.^23–26^ Among the new educational technologies in anatomy is the virtual anatomy dissection. A high-tech tool that allows students to perform some hands-on manipulation of a digital cadaver through an electronic screen in the form of a table. The use of Anatomage table in teaching anatomy lab sessions started 4 years ago in the faculty of medicine in Al-Balqa University. Anatomage Tables are used in teaching and learning anatomy during these three-hour weekly lab sessions.

This study is designed to evaluate the use of virtual anatomy dissection among a group of Jordanian medical students. The aim of this study is to determine the attitudes and perceptions of Jordanian medical students towards incorporating virtual dissection technology as part of their learning experience, thus testing the hypothesis that virtual dissection techniques are favored over traditional cadaveric dissection among medical students. The outcome of the study is expected to help and guide anatomists on the best ways of incorporating virtual dissection in their teaching to gain maximal students’ satisfaction and benefit.

## Methods

This study was carried out on medical students at a public Jordanian university, Al-Balqa Applied University, using a specially created anonymous questionnaire that investigates the students’ attitudes toward using virtual anatomy dissection; Anatomage Table (Anatomage Europe, Italy). Moreover, the questionnaire investigates the outputs of using Anatomage Table on the academic achievement of the medical students in comparison to the traditional methods used in anatomical education. Prior to the questionnaire’s dissemination, a pilot study involving 30 students was conducted to validate the questionnaire. The final version of the questionnaire was distributed on medical students via Google Forms in the computer rooms of the college of medicine at Al-Balqa Applied University. The first section of the Google Forms requested each participant to fill in informed written consent. Only those who provided the informed written consent were allowed to proceed and complete the questionnaire. The institutional review board (IRB) at Al-Balqa Applied University approved the study (Approval number: 19/2023).

The sample size was calculated using a sample size calculator based on a response distribution of 50%, a confidence level of 95%, and a margin of error of 5%. A sample size of 306 was appropriate. The actual sample size was higher as we had 414 respondents. For data analysis, JASP software (JASP software, Version 0.14.1; jasp-stats.org) was used. The collected data were gathered using Likert items (ordinal data), non-parametric tests were used for statistical analysis. Students’ responses in Likert scale (ie, strongly agree, agree, neutral, disagree, strongly disagree) were coded in numerical values (ie, 5, 4, 3, 2, 1, respectively) for the purpose of data analysis. Spearman’s rank correlation coefficient (*r*) was calculated to determine the presence and strength of correlations between different variables. Furthermore, the Mann–Whitney *U*-test was used to identify significant differences between the responses of male and female medical students. Moreover, the Wilcoxon signed-rank test was used to identify significant differences in within-group comparisons. The rank-biserial correlation was used to calculate the effect size in the last two mentioned tests. Significance level was set at a p-value < 0.05. Charts and tables were also used to illustrate the results.

## Results

The online questionnaire was sent to all medical students in the first, second, third, and fourth year of medical school, 753 students. The response rate was 55%, so the total number of participants n = 414. Of them, 297 (71.7%) were females and 117 (28.3%) were males. Most of the participants (360 (87%)) were first-year students. In addition, second-, third-, and fourth-year students also participated in the study but to a lesser extent (14 (3.3%), 31 (7.5%), and 9 (2.2%), respectively).

Table 1 shows that most students agreed that Anatomage Table helped them better understand and memorize anatomy lectures. Furthermore, they recommended Anatomage Table for other students to use it for learning Anatomy. Table 1 also shows that most students were interested in using this learning method in groups during lab sessions. Moreover, most of the students wanted to have access to Anatomage Table at any time. The data also revealed that students were interested in having instructors to guide them throughout using this learning tool. Of note, although students were highly satisfied with using Anatomage Table, they still believe that this tool cannot completely replace traditional practical sessions. The study also reported that Anatomage Table had more positive effect on the memorization and understanding of anatomy lectures among males than females. The results also showed that male medical students preferred and enjoyed using Anatomage Table more and were more interested in using it in groups.

**Table 1:** Students’ Attitudes Toward Anatomage Table as a Learning Avenue, and Their Attitudes Toward the Proposed Ways for Its Application

Question	Strongly Agree	Agree	Neutral	Disagree	Strongly Disagree	Mann–Whitney *U*-Test for the Significance of the Difference Between Males’ and Females’ Responses
I prefer Virtual Anatomy Dissection (Anatomage table) to replace traditional Anatomy practical sessions	97 (23.4%)	107 (25.8%)	73 (17.6%)	73 (17.6%)	64 (15.5%)	*p*<0.001, *r*=0.3 (Mean: F=3.1, M=3.7)
I enjoyed learning Anatomy through Virtual Anatomy Dissection	109 (26.3%)	125 (30.2%)	78 (18.8%)	55 (13.3%)	47 (11.4%)	*p*<0.001, *r*=0.3 (Mean: F=3.3, M=3.9)
Virtual Anatomy Dissection provides sufficient anatomical knowledge for medical students with no need for lectures	42 (10.2%)	67 (16.2)	101 (24.5%)	149 (49.2%)	54 (13.1%)	*p*=0.09, *r*=0.1 (Mean: F=2.7, M=2.9)
Virtual Anatomy Dissection help me understand anatomy lectures in a better way	145 (35.2%)	120 (29.1%)	64 (15.5%)	47 (11.4%)	36 (8.7%)	*p*<0.001, *r*=0.3 (Mean: F=3.5, M=4.2)
Virtual Anatomy Dissection help me memorize anatomical details in a better way	139 (33.7%)	125 (30.3%)	65 (15.7%)	50 (12.1%)	34 (8.2%)	*p*=0.004, *r*=0.2 (Mean: F=3.6, M=4.0)
Virtual Anatomy Dissection helped me increase my grades on the anatomy exam	69 (16.8%)	140 (34.1%)	123 (29.9%)	51 (12.4%)	28 (6.8%)	*p*=0.12, *r*<0.1 (Mean: F=3.3, M=3.6)
Virtual Anatomy Dissection in groups in the lab is interesting	159 (38.7%)	138 (33.6%)	49 (11.9%)	38 (9.2%)	27 (6.6%)	*p*=0.003, *r*=0.2 (Mean: F=3.8, M=4.2)
I prefer having an instructor to guide me through Virtual Anatomy Dissection	171 (41.3%)	162 (39.1%)	50 (12.1%)	19 (4.6%)	12 (2.9%)	*p*=0.49, *r*<0.1 (Mean: F=4.1, M=4.1)
I prefer to have access to the Virtual Anatomy Dissection at any time for self-learning	229 (55.9%)	127 (31.0%)	34 (8.3%)	9 (2.2%)	11 (2.7%)	*p*=0.34, *r*<0.1 (Mean: F=4.3, M=4.5)
I recommend using Virtual Anatomy Dissection for other students and other courses	139 (33.7%)	130 (31.5%)	71 (17.2%)	41 (9.9%)	32 (7.7%)	*p*=0.004, *r*=0.2 (Mean: F=3.6, M=4.1)

**Notes**: *p*<0.05 indicates a statistically significant difference between males and females in their attitudes toward the variable. 0.3< *r* <0.5 indicates a moderate effect size, and *r* less than or equal to 0.3 indicates a weak effect size.

**Abbreviations**: M, males; F, females.

Spearman’s rank correlation coefficient revealed that the positive effect of using Anatomage Table on students’ memorization and understanding of anatomy lectures was strongly and positively with the fact that students enjoyed anatomy sessions conducted using this tool of virtual anatomy dissection (*r* = 0.7 and *p* < 0.001 for both variables – ie, memorization and understanding). This shows the positive impact of this learning method in reducing student burnout that may occur during traditional educational methods.

**Figure 1:**
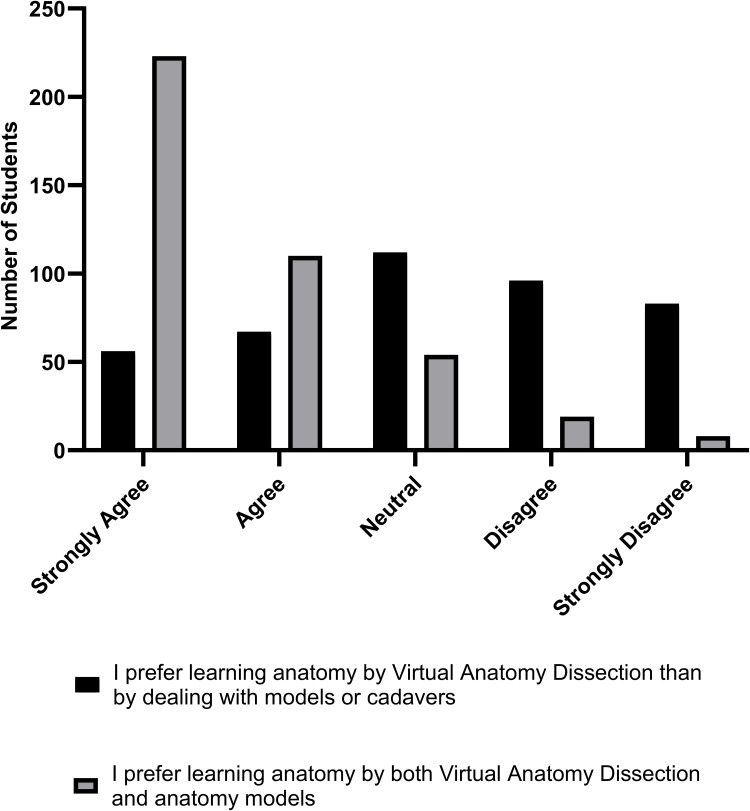
Students’ preference for the combined learning approach over the solitary use of virtual dissection-based learning. The figure shows students’ responses to the statements: I prefer learning anatomy by virtual anatomy dissection than by dealing with models or cadavers versus: I prefer learning anatomy by both virtual anatomy dissection and anatomy models.

Figure 1 shows that students prefer the combined learning approach over using only virtual dissection-based learning approach. Of note, Wilcoxon signed-rank test revealed a statistically significant difference between students’ preferences for both approaches, with the combined approach being a lot more preferred (*p*<0.001, *r*=0.9, Hodges-Lehman estimate = 2).

**Figure 2:**
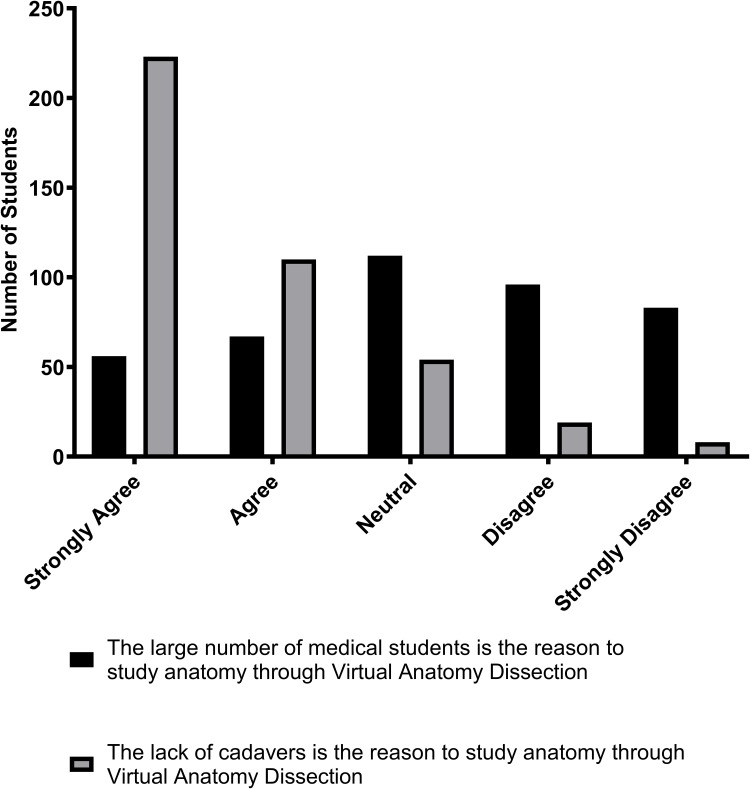
Reasons for students’ preference for the use of virtual anatomy dissection in learning anatomy. The figure shows students’ responses to the statements: The large number of medical students is the reason to study anatomy through virtual anatomy dissection versus: The lack of cadavers is the reason to study anatomy through virtual anatomy dissection.

Figure 2 shows plausible reasons for students’ preference for using virtual anatomy dissection in anatomical education. The two main reasons for using virtual anatomy dissection, as shown by the results, are the lack of cadavers and the large number of medical students.

## Discussion

The field of medical education is constantly evolving to meet the challenges of an ever changing world. The emergence of modern technology, changes in healthcare systems and the need for innovative teaching strategies are only a few of the factors contributing to medical education reform.^27^ Therefore, anatomy education has also experienced changes in order to remain aligned with updated medical curricula. With a multitude of changes occurring on a regular basis, feedback from students is considered essential to guarantee the successful reshaping of their educational experience. A student feedback survey conducted in 2021 to assess remote pre-clinical education provided insights as to its limitations and advantages, thus creating a framework around which sustainable remote learning curricula could be planned.^28^

The use of technology assisted teaching in anatomy is becoming increasingly evident, especially in the post-covid era.^29^ Virtual dissection technologies are, of course, expensive, and therefore not widely available in developing countries. So, while various studies have addressed the use of modern technology in medical education and found it to be extremely beneficial,^24–26^ the use of virtual dissection and its educational outcomes in low-to-middle-income developing countries have not been sufficiently studied.^30^ Accordingly, institutions cannot make the decision as to whether or not it is worth the cost to incorporate these new learning technologies as part of their teaching methods.

Results and feedback obtained from this study indicated a number of aspects in which the students favored the use of the Anatomage (virtual dissection) table Medical students of the 21st century have been named the “joystick generation” as they have a remarkable ability to understand and adapt to the use of high technology.^11^ Accordingly, they are more likely to prefer learning anatomy using tools such as the Anatomage table and give positive feedback regarding its use. At least 50% of the participants reported that use of the Anatomage table helped them improve their grades and almost 64% indicated that it helped them memorize anatomical details in a better way. These results are comparable to previous studies where the Anatomage table was found to increase students understanding of internal body organs and their relations.^31–33^

The current study also found that males noticed better memorization and preferred the Anatomage table more than females. This may be related to how males form memories based on their visual sense, as one study concluded.^34^ Another study also reported that when learning undergraduate physiology, males preferred visual learning.^35^ Research on learning styles preferred by students found that male students had a higher probability of choosing a unimodal learning style (Kinesthetic), whereas female students were more likely to use multiple learning styles consisting of visual, kinesthetic, and aural models.^36^

Additional results showed that students preferred working in groups and the Anatomage table provides students with the opportunity to discuss important topics. Group-based education provides a supportive network to share knowledge and validate clinical experience among learners.^37^ Students are also given space to voice their opinions and thoughts, which is the kind of verbal participation that contributes to active learning and student engagement.^38^

Use of the Anatomage table has multiple advantages that can assist students in their learning. It provides multiple visual applications that can enhance anatomy education, such as the observation of real patient radiological images and analysis of their pathologies.^31^ Furthermore, it is ideal to be used in distance learning conditions, similar to those faced both during the COVID-19 pandemic that restricted education globally.^39^,^40^ Another advantage of the Anatomage table is that it can be connected to storage devices, online platforms, or apps which allow students to access the images off campus.^31^,^41^

One of the most important and ongoing debates in anatomy education is the role of cadaveric dissection and the possibility of its replacement with technology-based teaching methods.^42^ This controversy is heightened in developing countries as there are further challenges to consider, namely, the large number of students in the face of a shortage of cadavers,^22^,^43^ the potential health hazards resulting from extensive exposure to certain chemicals like formalin,^34^ as well as the time and expertise required to prepare prosections.^42^ The positive feedback from students who have used the Anatomage table may present a sound argument that justifies its incorporation into the anatomy curriculum. This in turn may solve many of the issues that are being faced at the current time.

There are of course factors to be studied before such a drastic curricular change can be implemented. While research has shown that student performance is relatively equivalent when using cadaveric dissection or 3D visualization techniques,^42^,^44^ student perception and eagerness for learning must be taken into consideration. Participants in this study reported that use of the Anatomage table was interesting and enjoyable. Similarly, medical students at Case Western Reserve University were more enthusiastic about learning anatomy via the Anatomage table and clearly preferred it to the use of traditional cadaveric dissection.^44^ Despite the clear advantages of technology assisted teaching, it is the consensus among educators in the field of anatomy that the role of cadaveric dissection cannot be abolished entirely. Thus, the current trend leans more towards a combined method of teaching that includes both cadaveric dissection and technology assisted techniques.^11^

In conclusion, the Anatomage table is a powerful teaching method in undergraduate medical education, and its application in Al-Balqa Applied University has proven to be effective. While it may not be a replacement for cadaveric dissection, the Anatomage table can be used to help overcome the problems facing anatomical education in developing countries. This requires significant cooperation between universities and stakeholders to provide adequate funding and support for its incorporation into medical curricula.
